# Developing, implementing, and evaluating the visiting Neighbors’ program in rural Appalachia: A quality improvement protocol

**DOI:** 10.1371/journal.pone.0296438

**Published:** 2024-01-02

**Authors:** Ubolrat Piamjariyakul, Susan R. McKenrick, Angel Smothers, Angelo Giolzetti, Helen Melnick, Molly Beaver, Saima Shafique, Kesheng Wang, Kerri J. Carte, Brad Grimes, Marc W. Haut, R. Osvaldo Navia, Julie Hicks Patrick, Kirk Wilhelmsen

**Affiliations:** 1 West Virginia University School of Nursing, Morgantown, WV, United States of America; 2 West Virginia University School of Medicine, Morgantown, WV, United States of America; 3 Family & Community Development, West Virginia University-Extension, Kanawha County, Charleston, WV, United States of America; 4 Meredith Center for Career Services and Professional Development, West Virginia University College of Law, Morgantown, WV, United States of America; 5 Department of Behavioral Medicine/Psychiatry, Director, Memory Health Clinic, Rockefeller Neuroscience Institute, Morgantown, WV, United States of America; 6 Division Chief of Geriatrics, Palliative Medicine & Hospice and Grace Kinney Mead Chair of Geriatrics, West Virginia University School of Medicine, Morgantown, WV, United States of America; 7 Life-Span Developmental Psychology, West Virginia University, Morgantown, WV, United States of America; 8 Chief Cognitive Neurology, Rockefeller Neuroscience Institute, Morgantown, WV, United States of America; Utah State University, UNITED STATES

## Abstract

**Introduction:**

Older adults living alone in rural areas frequently experience health declines, social isolation, and limited access to services. To address these challenges, our medical academic university supported a quality improvement project for developing and evaluating the Visiting Neighbors program in two rural Appalachian counties. Our Visiting Neighbors program trained local volunteers to visit and guide rural older adults in healthy activities. These age-appropriate activities (Mingle, Manage, and Move– 3M’s) were designed to improve the functional health of older adults. The program includes four in-home visits and four follow-up telephone calls across three months.

**Purpose:**

The purpose of this paper was to describe the 3M’s Visiting Neighbors protocol steps guiding the quality improvement procedures relating to program development, implementation, and evaluation.

**Methods and materials:**

This Visiting Neighbors study used a single-group exploratory quality improvement design. This program was tested using quality improvement standards, including collecting participant questionnaires and visit observations.

**Results:**

Older adults (> 65 years) living alone (N = 30) participants were female (79%) with a mean age of 82.96 (SD = 7.87) years. Volunteer visitor participants (N = 10) were older adult females. Two volunteer visitors implemented each visit, guided by the 3M’s activities manual. All visits were verified as being consistently delivered (fidelity). Enrollment and retention data found the program was feasible to conduct. The older adult participants’ total program helpfulness ratings (1 to 5) were high (M = 51.27, SD = 3.77). All volunteer visitor’s program helpfulness ratings were also high (M = 51.78, SD = 3.73).

**Discussion:**

The Visiting Neighbors program consistently engaged older Appalachian adults living alone in the 3M’s activities. The feasibility and fidelity of the 3M’s home visits were verified. The quality improvement processes included engaging the expert advisory committee and rural county stakeholders to ensure the quality of the program development, implementation, and evaluation.

## Introduction

Rural West Virginia (WV) has the third largest population of adults 65 years and older in the United States [1]. Currently, 20.9% of the WV population is 65 years and older [2]. The population of older adults in WV is projected to become a quarter of the total state population by 2030 [3,4]. Thirty percent of the WV older adult population lives alone, and 70% of those who live alone are women [5,6]. Appalachia is a diverse mountainous region geographically spread across 13 states with 26.3 million residents. WV is the only state totally within the Appalachian region. Culturally, Appalachians are known to be independently minded, family and spiritually oriented [7,8] and have a lack of trust in ‘outsiders’ and healthcare systems [9,10]. In addition to the geographical mountainous challenges, Appalachians have extreme inequities in health and economic resources [11].

Similar to other states, West Virginia’s healthcare professional workforce is insufficient in size and scope for the medical, behavioral, and social support needs of the growing older adult population [12]. The existing workforce shortages of physicians, nurses, social workers, and caregiving workers will be exacerbated by the increasing number of older adults living longer with greater chronic healthcare needs [12]. Many older adults across these rural areas have limited access to healthcare services [13,14], suffer from social isolation and loneliness [15–17] as well as limited internet and cellular services [18,19]. Furthermore, one-third of WV older adults rely solely on social security as their primary source of income, while healthcare expenses remain their biggest financial burden [11,12].

In response to these challenges, the WV Academic Healthcare Innovation Summit was held in 2021 to identify the statewide health needs of older adults [20]. At the end of the summit, these multidisciplinary healthcare experts prioritized the Visiting Neighbors program for development, implementation, and evaluation. These experts recommended small funding for the 3M’s (Mingle, Manage, Move) Visiting Neighbors program. These 3 activities engage older adults in functional health per WHO guidelines [21,22]. The experts on the advisory committee and the rural stakeholders further developed these 3M’s activities for older adults who live alone because they are considered the most vulnerable [23,24].

A recent systematic review of studies supported the utilization of neighbors [25] and lay health workers in low-resource settings in preventing and controlling many illnesses [26]. Another systematic review of home visiting programs for older adults’ health found poor descriptions of programs limit the research [27]. Yet, a recent WHO report encourages program research because there is a gap in knowledge on the health status and function of older people [28]. Thus, to address these gaps, Visiting Neighbors volunteer-led home visits for the older adult population in rural Appalachia were designed to target maintaining health [25–28].

### Conceptual framework guiding the visiting neighbors intervention program

The conceptual framework used to guide the development of the Visiting Neighbors program was from the World Health Organization (WHO). The WHO action plan on healthy aging includes *“Aligning health systems to the needs of the older populations they serve* [29].*”* These volunteer-led 3M’s visits were envisioned as a possible option to reach the older adult population in rural WV [30–32]. WHO defined Healthy aging as *“more than just the absence of disease*.*”* Instead, health is maintaining the functional ability that enables well-being in older age [33,34]. Based on this concept of “functional health” defined by WHO [28], our volunteer visitors guide these older adults in the 3M’s healthy activities (*Mingle*, *Manage*, *and Move*). This quality improvement study engaged rural counties’ stakeholders in the Visiting Neighbors program. The study’s protocol steps based on quality improvement standards, are listed in the left column of Table 1. The center column lists the questions guiding the program evaluation. The right-hand column summarizes the quality improvement evaluation evidence aligned with the study’s specific aims. This quality improvement protocol aligns with the implementation science components of intervention development, implementation, and evaluation [35,36].

**Table 1:** Quality improvement standards for program development, implementation and evaluation.

Standards*	Development/Evaluation Guides	Evaluation Evidence per Program Specific Aims
**Visiting Neighbors Program Development**	What are the key assumptions underpinning the program?	• Defined healthy living activities (3M’s) based on the WHO definition of functional health (Aim 2)
	• What are the components of the intervention?	• Visiting Neighbors intervention manual and visitor training guides (Aim 2)
	• How is the program intended to be delivered?	• Fidelity guidelines for the second visitor to observe consistency of 3Ms delivery (Aim 2)
**Context**	• What are the characteristics of the service? • Environment in which the Visiting Neighbors Program is delivered?	• Multidisciplinary experts designed the 3M activities specific to elders living alone Aim 2) • Recruitment of visitors and older participants from the same neighborhood/county (Aim 1)
	• What are the broader conditional factors which impact key stakeholders’ experiences?	• Verified successful participant enrollment strategies using county seats, churches, and local nurses for older adults in rural Appalachia (Aim 1)
**Implementation**	• To what extent is the program implemented as intended over time and across settings?	• Conducting this in rural Appalachian, using quality improvement processes, verified feasibility of the program.
	• How do key stakeholders, including program developers, implementers, and older adult participants, participate in and respond to the intervention? • Are there any barriers and challenges to program implementation?	• Academic Healthcare Summit Advisory Working Group praised the implementation manual (Aim 2) • Volunteer Visitors engaged in Train-the-trainer sessions (Aim 2) Telephone contacts provided to older adults (Aim 1 & 2)
**Mechanisms of impact**	How do program components, persons, and contexts interact to influence program implementation and related outcomes?	• Implementation manual guides used (Aim 2) & visitor reports confirmed consistent delivery (fidelity) of 3M’s activities (Aim 2) impacted older adults’ engagement in the 3M’s (Aim 1) • Fidelity (consistent delivery) of the intervention was verified across participants (Aim 2) • The older adults and visitors highly rated the helpfulness of the program (Aim 3)
	• What generalizable lessons can be derived from the findings for implementing prevention and intervention programs?	• Having rural stakeholders (i.e., churches, health centers, social service agencies, and older adult volunteers) engaged in program development, enrollment, and implementation led to success (Aim 1 & 2) • Participants did recommend the Visiting Neighbors program to other older adults. (Aim 3)

### Purpose

This paper aims to report the testing of the 3M’s Visiting Neighbors intervention program. Further, the procedures, evaluation plans, preliminary results, and evidence from the program implementation are described. The specific aims of this study are to:

***Specific Aim 1*:** Describe the development of the 3M’s Visiting Neighbors intervention program and establish plans for the program implementation. Specifically, the feasibility of conducting the program for older adults living alone using volunteer visitors in the Visiting Neighbors program was tested. Older adults’ enrollment and retention are reported.

***Specific Aim 2*:** Evaluate the fidelity (consistency) of implementing the 3M’s program as designed. Strategies, materials, and the manual guiding 3M’s activities implementation are described. Visitor observers reported on the consistent delivery of the 3M’s activities.

***Specific Aim 3*:** Evaluate the program at the 3-month follow-up by (1) the older adult participants’ helpfulness ratings of the program and (2) rural volunteer visitors’ helpfulness ratings. Factors influencing the program’s impact are discussed.

## Methods and materials

### Design

This Visiting Neighbors program used a single-group exploratory quality improvement design. A quality improvement protocol guided the development, implementation, and evaluation of the program (Table 1) [37–39]. The program included four in-home visits and four follow-up telephone calls across three months. Two volunteer visitors conducted each visit, one engaged the older adult in the 3M’s activities while the second visitor was an observer. The observer followed the program implementation manual to ensure that the 3M’s activities were consistently delivered as designed.

The evaluation questionnaire rating the program’s helpfulness was completed by the older adult participants and the volunteer visitors who implemented the program. The evaluation questions (See Table 1, center column) provide evidence to determine if the Visiting Neighbors program might benefit older adults in rural Appalachia.

### Ethics statement

The Institution Review Board approved the Visiting Neighbor program procedures (IRB 2205583288) as a community quality improvement project. This quality improvement project used anonymous data collection from participants. All participants were informed about our program, including the IRB-approved description of the Visiting Neighbors program, the visits’ timeline, the risks and potential benefits, the small participant stipend, and the methods used to ensure confidentiality. Participants signed a consent to participate and safety guidelines for in-home visits agreements (for limited liability coverage recommended by the WVU legal office). Consent forms were filed separately from the data. All data sources (questionnaires) were de-identified and kept in a locked file cabinet in the research office. All the program staff completed the Collaborative Institutional Training Initiative (CITI) and strictly followed WVU’s privacy and confidentiality policies.

### Setting and participants

We enrolled a convenient sample of 30 older adult participants living alone in two rural counties in WV. This sample size (N = 30) is commonly acceptable for exploratory quality improvement studies [40]. Also enrolled in the study were volunteers to conduct the visits. There were 10 volunteer visitors who agreed to participate in the research training to observe and consistently deliver the 3M’s as designed. Per quality improvement standards, all ten volunteer visitors were invited to complete the program helpfulness evaluation rating scale after visits were conducted.

#### Eligibility criteria

Inclusion criteria were older adults (age ≥ 65 years) living alone in rural West Virginia (WV). Volunteer visitors were participants who agreed to complete training to deliver the 3M’s visits and provide confidential program helpfulness evaluation about the visiting sessions. Older adult participants were alert and oriented, provided written consent, and could read and write in English. This program enrolled older adults regardless of their socioeconomic status and health conditions. Exclusion criteria were older adults with a disability that precluded their use of Visiting Neighbors materials and those who live in nursing facilities (i.e., assisted living or nursing homes).

#### Recruitment and scheduling plan

*Recruiting Lead Visitors and Volunteer Visitors*. The two “lead visitors” from two rural counties were recruited to the program via connections with rural nurses in WV and personal contact by the project director. The lead visitor in one county was a social worker, and the lead visitor in the other county was a former in-home physical therapist. Each lead visitor compiled a team of older adult volunteer visitors living in their respective counties who agreed to be trained as “Visiting Neighbor” while also assisting with recruiting older adults. A total of 10 volunteer visitors engaged in the two training group sessions and implemented all the program visits. The volunteer visitors drove their car to the older adults’ home. The study provided a small stipend at $50/hour x 10 hours per each older adult.

*Recruiting Older Adults living alone in rural Appalachia*. Specifically, lead visitors contacted rural health providers’ offices, local churches, and centers for older adults in their counties to inquire about potential participants who met the inclusion criteria [41]. Also, enrolled participants identified other possible homes to visit. A key in recruitment was the guidance of the WVU School of Nursing Associate Dean for Community Engagement, based on her previous clinical care in rural areas and her county activities, such as her faith-based nursing program [42,43].

Two School of Nursing graduate research assistants were trained to conduct an initial telephone call to the potential participants to explain the program and assess their eligibility and willingness to participate. Once the older adults agreed to participate, their names and contact numbers (all kept confidential) were provided to the lead visitors who scheduled the home visits. Our graduate research assistants worked in the research office and were not involved in the 3M’s home visits. These graduate research assistants continued to track the participant scheduling plan and report the progress of enrollment, visit timelines, and retention quarterly. Retention efforts included these trained graduate nursing assistants’ weekly reminder calls to older adults. In addition, the older adult participants were given the research office’s telephone number and informed that they could leave messages or discuss any program concerns.

### Description of the Visiting Neighbors Program

The volunteer visitors were trained to deliver the Visiting Neighbors Program per the implementation manual. The program included four in-home visits, and four follow-up telephone calls across three months. The four visits promoted the 3M’s healthy functional living activities (Mingle, Manage, and Move). The first visit was an overall presentation of the program visits. The second ‘Mingle’ visit activities provided education and strategies to improve loneliness, social isolation, mental health, locating/utilizing local resources, and ways to stay connected with others. The third ‘Manage’ visit activities included nutrition and dietary counseling, medication management, handling household finances, and end-of-life-care option documents. The fourth ‘Move’ visit activities focused on staying active and multiple skills to safely exercise as one ages.

All 3M’s sessions were educational and included graphic illustrations to guide older adults to practice the 3M’s skills. Visitors used demonstration and empirically verified coaching techniques in each home visit [44]. There were pictorial demonstrations of the activities in the manual. Older adults did a “teach-back” demonstration showing the visitors the 3M’s activities learned at that visit. Older adults were encouraged to practice the activities independently and were given a log to track their 3M’s use. Telephone follow-up calls reinforced ongoing practicing of the 3M’s healthy living activities, utilizing local resources, and contacting social support and health care providers. The follow-up calls also provided the opportunity for participants to ask questions.

Notably, the details of each of the 3M’s activities were created by educational health experts (older adult health educators from the WVU School of Medicine, Departments of Behavioral Medicine and Psychiatry, Gerontology, School of Nursing, and rural home visit nurses). These experts established the content validity of the program. The 3M’s manual was designed by rural home care nurses and two (Master’s level) students. The manual guides implementation steps and consistent delivery (fidelity) of the 3M’s to each older adult. The Visiting Neighbors manual includes the handout guides, pictures of healthy food, safe exercise, and a list of resources for social support associated with 3M’s contents [45]. Each home visit was designed for approximately 2 hours, and the follow-up telephone calls were 15–30 minutes long.

From the program initiation, several rural stakeholders (i.e., churches, health centers, social service agencies, and older adult volunteers) were engaged in the Visiting Neighbors program development and implementation. Prior to the program, the implementation manual was reviewed by an older adult to ensure that handouts for each activity were easy to read and follow. Thus, the Visiting Neighbors program’s content and face validity were established.

### Visitors training for delivery of the program

All ten visitors were trained to use the Visiting Neighbors 3M’s implementation manual. Training sessions were provided with teaching, examples, and practice of each 3M delivery via the manual. The 3M’s visit manual and PowerPoint video recording were used to train the visitors for each visit. Visitors also engaged in role-playing to prepare for each visiting session. For quality assurance, a video and training sessions on appropriate communication techniques and following the 3M’s visit manual were provided to the visitors. Specifically, all visitors were taught to use coaching (positive directions or encouragement) and teach-back methods *(“please describe or show me what you learned today”*) to ensure the older adult participant’s understanding of each 3M’s session [42,44].

### Procedures for Visiting Neighbors Program implementation

Two local volunteer visitors residing in the same rural area (or county) delivered the program. The visitor would telephone one day before each home visit as a reminder. During the first home visit, the visitors explained the objectives of the Visiting Neighbors program. Also, the Visitor explained and obtained the participant’s signed informed consent and limited liability coverage form recommended by the WVU Legal Office. Older adults were informed they would receive a cash card of $100 for their time across the visits.

There were two visitors at each visit, one to implement the 3M’s activities and the other to observe. The observer visitor used the manual guidelines to observe the 3M’s instruction accuracy and consistency of delivery. Each visitor reported the visit’s fidelity implementation observations and any comments or requests from the older adults to the research office. The GRA tabulated the fidelity observation (yes/no) and an anonymous list of all comments or requests from the older adults. The project director monitored these observation lists following each visit. Comments and requests were used to assist in improving our future programs. Thus, the project director monitored the fidelity across the study timeline to ensure the reliable and valid implementation of the Visiting Neighbors program [46]. At each subsequent visit, the visitors reviewed the previous visit’s content and 3M’s activities and provided reinforcement or demonstration, if needed. At each visit, the visitors allowed sufficient time for participants to practice the 3M’s activity skills and ask questions. The date and time of the next visit were written down on the participant’s home visit calendar.

### Data collection

Questionnaire data were collected from each older adult participant at baseline on sociodemographic variables. Then, following the completion of the program visits, the program helpfulness ratings were collected from both older adults and volunteer visitors. The program evaluation helpfulness scale was collected anonymously and placed in a closed envelope by the older adults at the end of the last home visit. All data collected were kept with only the ID code. The data manager entered data into a secured, password-protected *Qualtrics* database stored at WVU Health Sciences Center. The SPSS (version 29) was used for data analysis [47].

#### Measures and materials for the Visiting Neighbor Program

The following two measures were used to gather data. These standardized questionnaires had established validity and reliability and were previously used with older adults.

*Sociodemographic variables*. Participants were asked to provide sociodemographic information, including age (in years), gender, race/ethnicity, level of education, marital status, county of residence, and income adequacy. Participants were also asked how long they have lived alone and if they are currently employed. This measure has been used frequently and found valid in other studies [36,42].

*Helpfulness Rating Scale*. The 11-item Likert-type helpfulness rating scale was used to rate the helpfulness of the 3M’s program by older adults and visitors [44,48,49]. One sample item was “Overall, the Visiting Neighbors Program was helpful in keeping me healthy.” Another sample item was, “I was able to select better food or nutrition that was good for my health.” Another item for the older adults and visitors was, “I would recommend the Visiting Neighbors program to other older adults if available in their communities.” Response options were rated on a 5-point Likert scale, from 1 (strongly disagree/not helpful) to 5 (strongly agree/very helpful). A higher score indicates greater helpfulness of the program.

Further, the volunteer visitors rated three additional items: “The program is likely to be acceptable by older adults for future use,” “I was able to follow the Visiting Neighbors manual and its sections,” and “The Visiting Neighbors implementation manual included 3M’s that were all key healthy living activities for older adults. This measure has years of frequent use in program evaluation [48].

### Data analyses and evaluation plans

For Specific Aim 1, feasibility was evaluated from participants’ enrollment and retention. For Specific Aim 2, the visitors reported observations to determine consistent 3M’s delivery (program fidelity). For Specific Aim 3, after completing the home visits, older adult participants and volunteer visitors completed the program evaluation helpfulness scale.

## Results of the quality improvement program

Quality improvement standards and processes were used in program development, implementation, and evaluation (See SQUIRE checklist, Supporting Document). The quality improvement standards are summarized in the left-hand column of Table 1. The questions in the centered column guide the development and evaluation of quality improvement programs. And the evaluation evidence aligned with the study’s Specific Aims are summarized in the right-hand column. The evidence indicated quality improvement standards were addressed via the program development, context, implementation, and impact.

### Sociodemographic results

Within six months, 62 older adults were screened for enrollment, 30 were enrolled in the program (19 did not meet the inclusion criteria, i.e., did not live alone), and 13 refused to participate. Of the 30 enrolled, participants were predominantly White females (79%), and the average age was 81.40 (SD = 8.18) years old. On average, participants lived alone for 18.17 years (SD = 15.05). Most participants (n = 19, 63.3%) completed high school or less, and 73% (n = 22) were widowed. Twenty-nine participants (96.67%) were unemployed, and two were disabled. Participants (n = 29, 96.67%) had Medicare, one had employment-based, and 19 had additional Medicaid or other insurance coverage. Of 30, twenty-four older adults (80%) reported sufficient income (a little extra or always have money left over), while six (20%) reported income insufficient (can’t make ends meet or just enough). All ten volunteer visitors were White, older adults (age over 60+), except two who were middle-aged. See Table 2.

**Table 2:** Older adult participants sociodemographic characteristics (N = 30).

Characteristics	N (%)
**Gender** n (%)	
Male	6 (20)
Female	24 (80)
**Ethnicity** n (%)	
Non-Hispanic	30 (100.0)
**Race** n (%)	
Black/African American	1 (3.33)
White	29 (96.67)
**Education** n (%)	
High school or less	19 (63.33)
Some college	6 (20)
Completed college or more	5 (16.67)
**Marital status** n (%)	
Widowed	22 (73.33)
Divorced	4 (13.33)
Never married	4 (13.33)
**Employment**	
Employed	1(3.33)
Unemployed	29 (96.67)
**Insurance**	
Medicare	29 (96.67)
Private employment-based	1 (3.33)
Additionally, Medicaid and other insurance	19 (63.33)
**Income level**	
I can’t make ends meet	4 (13.33)
I have just enough; no more	2 (6.67)
I have enough, a little extra sometimes	14 (46.67)
I always have money left over	10 (33.33)
**Age,** Mean (SD)	81.40 (8.18) years Range 66 to 96
**Living alone time,** Mean (SD)	18.17 (15.05) years Range 1 to 54

#### Feasibility results (Aim 1)

There was successful enrollment of 30 older adults as planned. All older adults except one completed all 3M’s (4 home visits and 4 telephone follow-up visits). One participant could not complete the final visit at 3-months due to moving out of state. All visitors reported that the 3M’s training manual was helpful, the visits were enjoyable, and the program engaged the older adults in the 3M’s activities. Thus, the feasibility of developing and implementing the program was verified.

#### Fidelity results (Aim 2)

Ten volunteer visitors from the two rural WV counties were trained for home visits and consistent delivery of the 3M’s Visiting Neighbors program. Across the home visits, visitors reported no difficulty following the 3M’s Visiting Neighbors training manual. The fidelity observations by the visitors verified that the delivery across all visits was consistent per the manual.

#### Helpfulness evaluation results (Aim 3)

Overall, the 11-item helpfulness scale (Table 3) indicated the older adult participants (n = 29) were satisfied with the Visiting Neighbors program. All older adult participants rated the program (on the scale of 1 to 5) and home visits highly helpful. Scores on each item ranged from 4.34 to 5.0. The total helpfulness scale score was high (M = 51.27, SD = 3.77). Notably, the item rating the impact or helpfulness of the program on loneliness and depression was positively high. Both visitors and older adults stated they would recommend the program to other older adults if available in their areas.

**Table 3:** Helpfulness scale responses by volunteer visitors and older adults.

Helpfulness Scale Items (Response Options 1 to 5) *Items 1 to 11 were answered by Volunteer Visitors and Older Adults* *(1 = strongly disagree/Not Helpful; 5 = agree/very helpful)*	Volunteer Visitors (n = 9)		Older Adults (N = 29)	
	M	SD	M	SD
1. The Visiting Neighbors program helped increase [participant] activity level.	4.78	0.44	4.57	0.56
2. The Visiting Neighbors program helps [participant] be aware of and manage negative feelings (i.e., loneliness, depression, or anxiety).	4.78	0.44	4.34	0.84
3. [Participant] is able to select better food or nutrition that is good for his/her health.	4.33	0.87	4.55	0.63
4. [Participant] feels like the Visiting Neighbors program helps manage [his/her] affairs (medications, conditions, ADLs) better now.	4.56	0.53	4.34	0.80
5. The Visiting Neighbors discussion about available resources and/or home assistance in [participant’s] community to support aging at home was useful.	4.67	0.50	4.55	0.69
6. It was helpful having a nurse (trained health care staff) provide education about [participant’s] chronic health conditions (hypertension, Diabetes, hyperlipidemia).	4.33	0.87	4/75	0.40
7. The explanation of the Advance Directive was helpful.	4.67	0.50	4.68	0.38
8. The Visiting Neighbors nurse and visitor gave [participant] time to ask questions.	5.00	0.00	4.83	0.38
9. [Participant] was comfortable during the discussion with the nurse and visitor.	5.00	0.00	4.86	0.35
10. Overall, the Visiting Neighbors program is helpful in keeping [participant] stay healthy.	4.78	0.44	4.79	0.41
11. [Participant] would recommend the Visiting Neighbors program to other older adults if available in their communities.	4.89	0.33	5.00	0.00
*Items 12 to 14 were answered by volunteer visitors only*.				
12. The Visiting Neighbors program is likely to be acceptable by older adults for future use.	4.89	.033	--	--
13. The visitor was able to follow the Visiting Neighbors manual and its sections.	4.89	0.33	--	--
14. The Visiting Neighbors manual included 3M’s and key healthy living activities.	4.89	0.33	--	--

Nine of 10 volunteer visitors completed the program helpfulness scale. These volunteer visitors rated the program and home visits highly helpful to each older adult. The visitors’ mean scores on each item ranged from 4.33 to 4.78. The visitors’ total helpfulness score was high (M = 51.78, SD = 3.73). At the end of the helpfulness scale, visitors also rated additional items: if the program would be acceptable to older adults in the future if the visitors were able to follow each section of the implementation manual, and if the Visiting Neighbors manual clearly illustrated each of the 3M’s healthy living activities. The mean score of these three items was a high rating (M = 4.89, SD = .33).

Helpfulness of the visits is reflected in the visitors’ brief notes, including their positive verification of implementation fidelity. For example, the older adults described how the 3M’s activities helped them remain in their homes. The visitors’ notes also listed concerns or requests from these older adults. Most of the concerns were about their faith in God, loss of loved ones and friends, financial hardship and the need for transportation.

## Discussion

In summary, this study addressed the aim of engaging rural Appalachian stakeholders in quality improvement protocol to develop and test the Visiting Neighbors program. The context and rationale for the Visiting Neighbors program was based on the growing older population, their healthcare needs, and other challenges of living alone in remote rural areas of WV and the healthcare workforce challenges [12,50]. We also described the background for the program’s development, implementation, and evaluation guided by the quality improvement standards.

The initial findings were related to the study Specific Aims 1, 2, and 3. Results indicated successful program enrollment (N = 30), completing all visits (except one older adult), and data collection. Thus, the feasibility of developing and implementing this Visiting Neighbors Program was verified (Specific Aim 1). Enrollment was achieved by the helpful strategies of the local Appalachian stakeholders, local faith-based social network, and rural health senior centers. Neighbors helping neighbors was helpful and acceptable in rural settings [30–32]. Other researchers have also found that enrollment and implementation by trained local visitors familiar with the Appalachian culture helped develop trust [10,41]. For Specific Aim 2, visitors reported that the Visiting Neighbors Program 3M’s delivery was consistent (high fidelity) across participants. As per Specific Aim 3, older adult participants and volunteer visitors rated the 3M’s program components as helpful. The older adults voiced that they benefited from the home visits and recommended continuing the program. The participants agreed they would recommend the program to other older adults if available in rural areas.

The mechanisms of impact that were described by the visitors as making the program acceptable to the older adults was the 3M’s information. The participants highly rated the information on safe daily activities, nutrition, and mental health. The visitors reported that the program impacted older adults’ self-confidence to communicate with their healthcare providers. The 3M’s healthy activities were designed to promote functional health in older adults [21,22]. Visitors stated their training, practice, and the step-by-step guide manual were important to consistent 3M’s delivery. Also, an impactful factor was the trust and rapport established with the older adults by local visitors [9,10,25]. The engagement of county stakeholders in program development facilitated participant recruitment. Another technique that helped to build trust with participants was sharing the office telephone number with the opportunity to call for any concerns. An anonymous voicemailbox in future studies would allow older adults to share their feedback freely.

During the program implementation, some barriers were reported. For the enrollment, some families were skeptical due to worries about being reported to authorities for unsafe conditions or lack of necessities (such as food, water, and heat). Such reports to authorities might lead to removing older adults from their homes. Yet, recruitment/enrollment was accomplished in a timely manner. The visitors’ notes revealed the older adults’ concerns about long-term feelings of loss, loneliness, financial difficulty, and transportation needs. Thus, it is recommended that coping with loss, palliative, and mental health care be added to the 3M’s, along with referrals to resources for financial and transportation needs. Another challenge was the winter in rural West Virginia, affecting visitors’ travel conditions, which delayed some visits. However, rescheduling was done at the participant’s convenience. Thus, the flexibility of home visit schedules contributed to enhancing the program’s implementation.

A few limitations of the study were noted. Minority participants were underrepresented, as is the population of the state of WV, and these two participating counties are 97.2% and 93.8% white [2]. To improve future testing of the study, including the few minority areas in rural Appalachian counties should be sought. Concerns about sustaining this volunteer program without administrative support have been discussed. There are recommendations for continuing the program by county rural stakeholders (i.e., churches, health centers, social service agencies). Reporting on the older adults’ health was also suggested to improve the Visiting Neighbors’ future programs. Also, studies comparing 3M’s visits with groups receiving only information or with other programs should be undertaken. A larger study with a control group is warranted.

## Conclusion

The 3M’s Visiting Neighbors program, designed by multidisciplinary healthcare experts and older adult health educators, was implemented by local volunteers. All the older adult participants could follow the 3M’s healthy activities demonstrations. Feasibility and fidelity of the Visiting Neighbors program implementation were documented. Overall, the program was highly evaluated by older adult participants and visiting neighbor volunteers. Discussion on the resources needed for the Visiting Neighbors Program to be transferable and scalable to other counties in WV is recommended. The Visiting Neighbors program could be a resource for rural older adults who live alone.

## Supporting information

S1 Checklist Reporting checklist for quality improvement in health care. (DOCX)

## Abbreviations

IRB: Institutional Review Board
WV: West Virginia
3M’s: Mingle, Manage, Move
QoL: Quality of Life
